# The spatiotemporal dynamics of COVID-19 in Europe: time-series clustering maps 5 distinct trajectories to spatial patterns

**DOI:** 10.1186/s12963-025-00405-w

**Published:** 2025-08-05

**Authors:** Sarah Habershon, Kolja Nenoff, Guido Kraemer, Lennart Schüler, Heinrich Zozmann, Justin M. Calabrese, Sabine Attinger, Miguel D. Mahecha

**Affiliations:** 1 Institute for Earth System Sciences and Remote Sensing, Talstraße 35, 04103 Leipzig, Germany; 2 Helmholtz Centre for Environmental Research, Leipzig, Germany; 3 CASUS - Center for Advanced Systems Understanding, Helmholtz-Zentrum Dresden-Rossendorf e.V. (HZDR), Dresden, Germany

## Abstract

The COVID-19 pandemic affected Europe unevenly, with surges in infections and deaths fluctuating across different regions and time periods. Hyper-localised hotspots and staggered timelines created intense, asynchronous waves of infections and deaths that distort country-level and cumulative data, obscuring the pandemic’s spatiotemporal dynamics through aggregation. Despite extensive research comparing states and analysing subnational variance within individual countries, the detailed subnational and transnational dynamics of the COVID-19 pandemic across Europe as a whole have not been comprehensively described. Here we show that time-series clustering, applied to weekly excess mortality estimates for subnational NUTS3 administrative regions of 27 countries in Europe, identifies five distinct pandemic trajectories which map to spatial patterns. The trajectories comprise two subgroups, representing contrasting pandemic dynamics in eastern and western Europe. Western Europe exhibits concentric arrangements of mortality impact, with secondary and tertiary impact zones surrounding outbreak epicenters. Eastern Europe exhibits internally homogeneous spatial dynamics, possibly due to the deferral of the first major mortality wave.

## Introduction

The COVID-19 pandemic in Europe exhibited complex spatiotemporal dynamics. Fluctuating surges in infections and deaths across different regions and timeframes made its highly variable mortality impact difficult to describe and interpret [6, 33, 35, 40, 46, 47].

Previous studies have explored the COVID-19’s spatiotemporal dynamics both within and outside Europe, demonstrating that time-series trajectory clustering is a useful approach to understanding COVID-19’s spread and impact over time [12, 37, 41, 52]. Cluster analyses of European states and regions identify spatial groupings which exhibit similar trajectories, but are largely conducted using data from bespoke COVID-19 data collection systems which vary across the continent, aggregated to a high level, and covering few countries. Their conclusions prove the spatiotemporal clustering concept in principle but lack the required resolution, coverage, and comparability to describe the pandemic’s spatiotemporal dynamics, in detail, across Europe.

Two key factors characterise the challenge this research gap presents: the lack of high-quality comparable data, and the spatiotemporal scale at which analyses are performed.

Understanding COVID-19’s impact in Europe, which is the world’s most internally and globally connected region [5], requires trans-national analyses of subnational data at high spatiotemporal resolution. However, most trans-European analyses use case rates or confirmed deaths from COVID-19, metrics which are not consistent or comparable between or even within countries [50].

Case rates are heavily influenced by the availability of tests, and differing testing and reporting policies. Mortality may appear to be a more reliable metric, but differences in the ways COVID-19 deaths are defined, counted, and reported compromise comparability. In some countries such as Germany and France, deaths from COVID-19 are recorded on the basis of a clinical diagnosis. In other countries such as Austria and Italy, a positive laboratory test is enough to classify a death as a COVID-19 death, which leads to inconsistencies deriving from testing availability and criteria, and may also include false positives, such as cases where a patient contracted COVID-19 in the hospital after presenting with a terminal injury [32]. Some jurisdictions also changed their definitions over time. In March 2020, Denmark changed its definition from any death occurring within 60 days of a COVID-19 diagnosis, to one occurring within 30 days [44].

We address the problem of inconsistent data and definitions by using routinely collected all-cause mortality data to estimate excess mortality. This approach has the advantage of consistency: all-cause mortality is less sensitive to misclassification or inconsistent definition than the bespoke data collection and reporting systems established during the pandemic [8]. It also captures the pandemic’s indirect mortality impact, making it a more comprehensive indicator.

The second major analytical challenge is the unit of analysis. The pandemic’s spatial dynamics are characterised by subnational ‘hotspots’ of elevated mortality impact both within countries and straddling international borders [11]. Because of these dynamics, the nation-state is not a useful unit of analysis. High-level spatial aggregation obscures internal variance in outcomes within states [10], fails to differentiate ‘spatially asynchronous waves’ (see Harvey et al. [28]) and introduces aggregation bias [13]. Similarly, using cumulative or temporally aggregated data conceals the intensity of the pandemic’s hyper-localised waves and its progress across the continent over time [28, 34].

We address the need for trans-national, high-resolution analysis by estimating weekly excess mortality for NUTS3 administrative regions of 27 countries in Europe: the highest spatiotemporal resolution for which all-cause mortality data is available. We cluster the weekly excess mortality trajectories of 770 NUTS3 administrative units to identify and describe spatiotemporal dynamics of COVID-19 mortality impact in Europe. Using this subnational, trans-European variable allows for much more granular analysis than any previous study, covering a larger geographical area at higher resolution over a longer time period.

## Methods

### Excess mortality

Excess mortality is recommended by both the European Centre for Disease Prevention and Control [19] and the World Health Organisation [53] as the best metric for understanding and comparing COVID-19 mortality impact.

We use weekly excess mortality to measure COVID-19 mortality impact, derived from routinely collected administrative data, at the third level of the EU’s Nomenclature of Territorial Units for Statistics (NUTS3). NUTS3 refers to the EU’s smallest administrative regions, for instance départements in France, provincias in Spain, or arrondissements in Belgium. We selected excess mortality over more comprehensive indicators such as age-standardized death rate or life expectancy change, because although weekly deaths are available for many countries by sex and age-group, the further disaggregation introduces inconsistencies in coverage. Additionally, the disaggregated values for NUTS3 units are so small that using the age breakdown risks amplifying errors and noise. Ultimately, the simple calculation required for the excess mortality rate enables easy reaggregation, making it the the best fit for the purpose of this analysis.

### Data preparation

The excess mortality for each NUTS3 unit is estimated as the percentage difference between the weekly all-cause mortality count in 2020 and 2021 and the mean mortality count in the corresponding week during the period 2015–2019. No age stratification or population data is included: the total mortality burden is the metric of interest.

Excess mortality is calculated as

$$\begin{aligned} m_e = \frac{d - \hat{d}}{\hat{d}} \end{aligned}$$

where $m_e$ is excess mortality, $d$ is the raw number of deaths and $\hat{d}$ the expected number of deaths. All values are calculated separately by week and NUTS3 region. $\hat{d}$ is calculated as the mean mortality over the years 2015–2019 in the same region and the same week of the year.

Weekly all-cause mortality data is sourced from Eurostat’s *exceptional data collection on total weekly deaths*, at NUTS3 level (Eurostat [20]). Deaths are reported by their date of occurrence. Sweden reported 5,614 deaths during the study period for which the date is unknown – these are excluded from the analysis. Estonian data from 2000–2019 is under the old NUTS-2016 classification, and 40 units in Belgium, Norway, and Spain have been redrawn since 2015. These instances are recoded, where possible, to their corresponding 2021 codes. Germany does not supply weekly all-cause mortality at NUTS3 level to Eurostat and is therefore excluded entirely. The final data set consists of weekly excess mortality trajectories for 770 NUTS3 units of Austria, Belgium, Bulgaria, Switzerland, Cyprus, Czechia, Denmark, Estonia, Greece, Spain, Finland, France, Hungary, Italy, Liechtenstein, Lithuania, Luxembourg, Latvia, Montenegro, Netherlands, Norway, Poland, Portugal, Romania, Serbia, Sweden, and Slovakia from 2020 to 2021.

### Selecting a clustering method

We tested and compared three clustering methods, *k*-Nearest Neighbours (*k*-NN), Affinity Propagation (AP) [21], and hierarchical agglomerative clustering with Ward’s minimum variance method on Euclidean distances [31]. *k*-NN is the most established method, and more frequently cited, but is sensitive to outliers and noise; this is a known limitation [54].

The weekly excess mortality data we use contains outliers, such as the NUTS3 units in northern Italy and central Spain, where weekly excess mortality exceeded 600% during March 2020. The data may also be affected by inconsistencies in reporting standards, quality, and timeliness.

We cluster the NUTS3 time series without considering any geographical information. Only excess mortality as proportion of the expected mortality goes into the analysis, in order to not prescribe any pattern. The NUTS3 units are taken as observations, and weeks as variables for the clustering.

To test the *k*-NN approach [27], we used the Within-Cluster-Sum of Squared Errors (WSS) to establish the optimal *k* value. We plotted the diminishing WSS (see Fig. 4 in Appendix Method comparison and robustness assessment) for increasing numbers of clusters, and identified 5 as the point at which the explanatory value added by additional clusters became marginal, rather than meaningful.

To address the limitations of *k*-NN and hierarchical agglomerative clustering, both of which are sensitive to outliers due to their reliance on Euclidean distance, we selected the AP method. Because the AP algorithm considers the whole dataset, it is less sensitive to noise and outliers. This advantage was apparent in the results.

#### Clustering with affinity propagation

Affinity Propagation is used as fully data driven clustering approach. The method identifies clusters based on pairwise similarities (or dissimilarities) between data points, using a message-passing approach to recursively update the likelihood of each trajectory being the exemplar for another. It identifies one exemplar trajectory per cluster, and assigns each data point to the cluster of its most suitable exemplar. AP dynamically determines the number of clusters based on the data, but the quantile parameter (*q* value) influences the number of clusters it will identify. Each data point is assigned a preference value (*p*) which indicates how likely it is to be chosen as an exemplar. The *q* value specifies which quantile of the similarity distribution is used to determine these preference values. We established that $q = 0$, which is the minimum similarity value and reduces the probability of each data point being selected as an exemplar, yielded 5 clusters. Based on the WSS, we determined that this was the optimal sensitivity. We used the apcluster package (version 1.4.11) [9], running on R version 4.4.1.

When we generated 5 clusters from the data using AP with the damping factor *q* = 0, we achieved broadly similar results to the *k*-NN analysis (see Fig. 5).

#### Robustness assessment

To assess the robustness of the clustering, we compared the AP results to an additional clustering method: hierarchical agglomerative clustering. When the hierarchical method was applied, 124 out of 770 NUTS3 units were reassigned.

We concluded that the three methods delivered a relatively high degree of consistency in the overall structure of the clustering. It was evident from the distribution of units between the clusters (see Appendix Method comparison and robustness assessment) that the AP approach was less sensitive to the outliers in the data, and produced more useful and intuitive clusters. The *k*-NN clustering approach yielded one cluster containing only 15 units; in the hierarchical clustering approach this cluster contained only 12 units. Clustering with AP increased the size of this cluster to 27 units. We selected the output of the AP algorithm for our results.

**Fig. 1:**
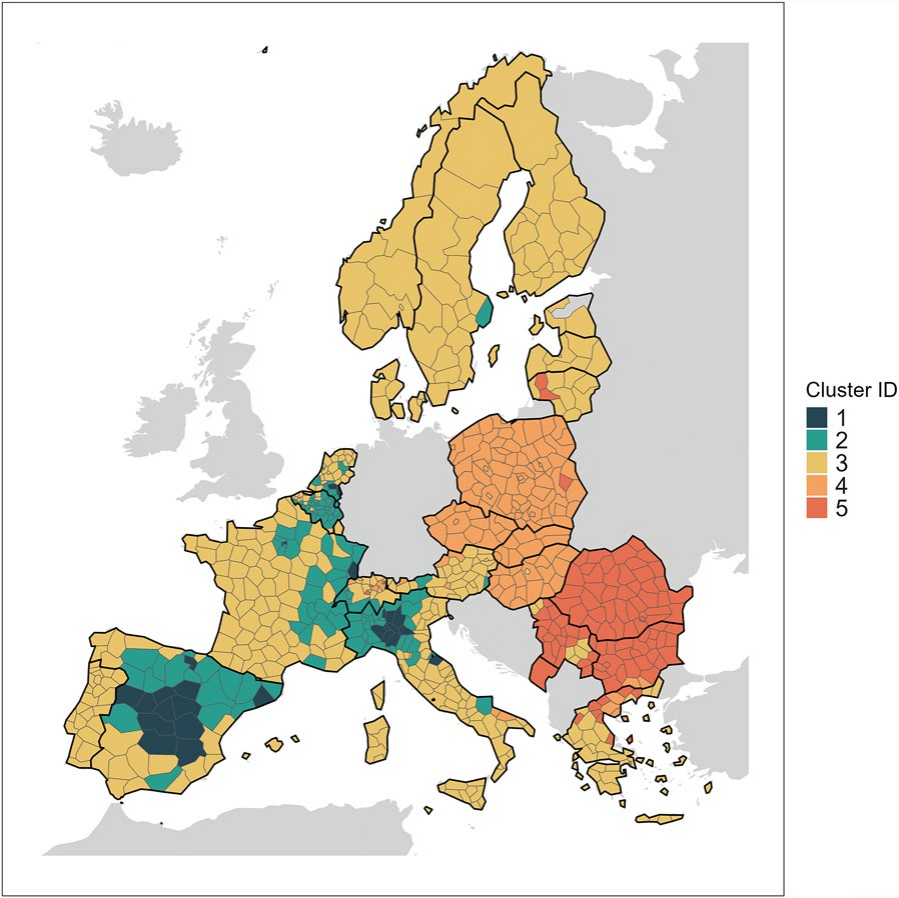
NUTS3 administrative units of Europe clustered by their excess mortality trajectories during the COVID-19 pandemic years 2020-2021, using Affinity Propagation. Countries which do not report weekly all-cause mortality at NUTS3 level are excluded

## Results

### Time series clustering reveals corresponding geographical groups

Affinity propagation clustering of the weekly excess mortality trajectories in 770 NUTS3 units across Europe identified 5 cluster groups. These groups reveal geographical patterns (see Fig. 1) although no geographical information was included in the clustering process.

- Cluster 1 (*n* = 27), comprising major western European cities and their immediate surroundings (Paris, Madrid, Barcelona, Milan), plus a handful of others such as Pesaro and Urbino in north-East Italy, Brabant and Limburg on the internal Dutch border, Alava in northern Spain, and Haut Rhin on the eastern border of France.
- Cluster 2 (*n* = 123) comprises the areas surrounding the Cluster 1 centres (i.e. around Madrid, Paris and Milan), Stockholm, and most of Belgium.
- Cluster 3 (*n* = 390), representing western France, the north and south coasts of Spain, Portugal, most of the Nordic and Baltic states, southern Greece, and the southeast of Italy.
- Cluster 4 (*n* = 129), comprising almost the complete entirety of Poland, Czechia, Slovakia and Hungary, as well as a handful of Swiss cantons.
- Cluster 5 (*n* = 101), comprising almost the entirety of Romania and Bulgaria, Serbia and Montenegro.

**Fig. 2:**
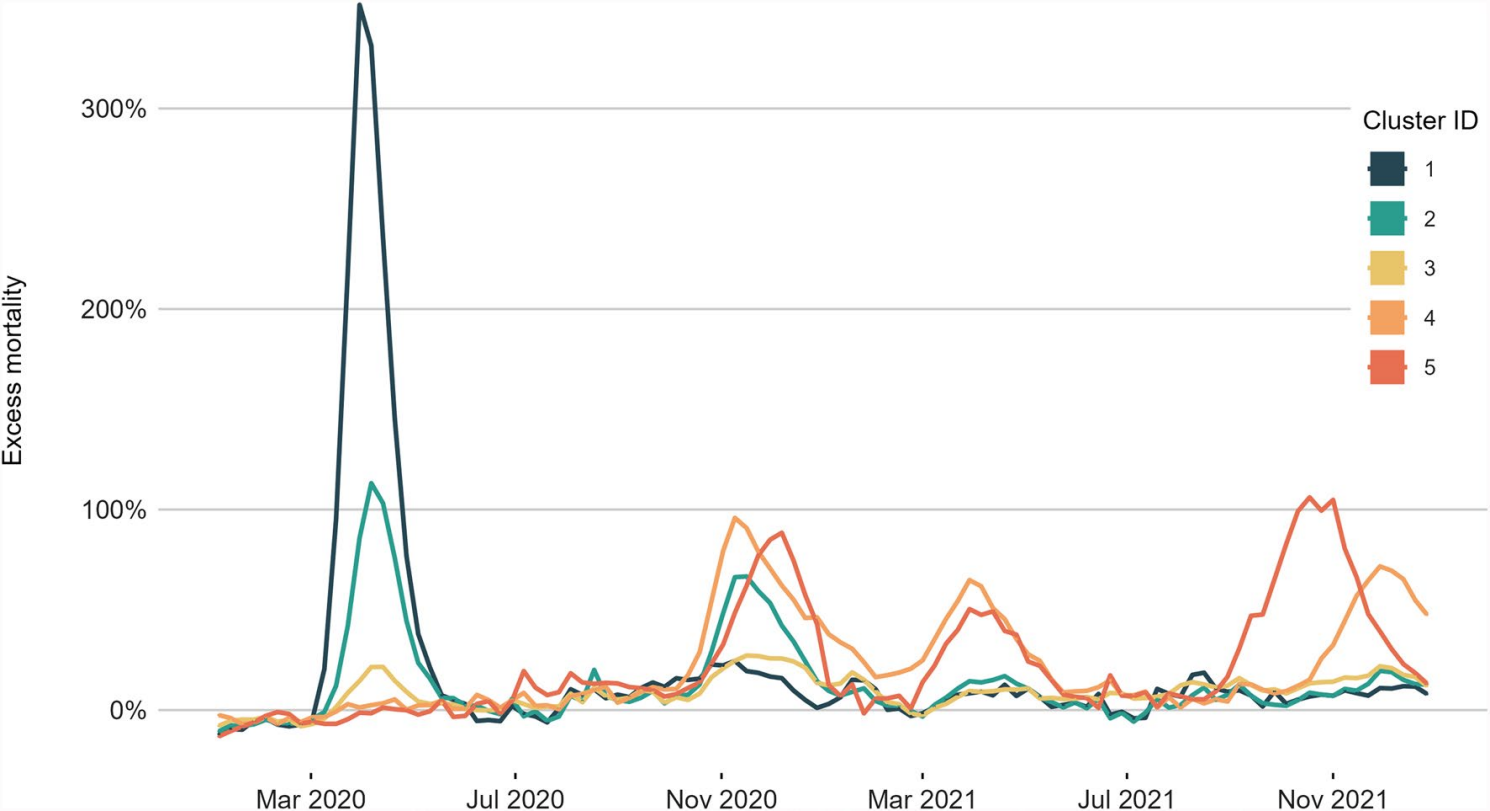
Overall weekly excess mortality during the COVID-19 pandemic years 2020–2021 within the clusters as shown in Fig. 1

There is a clear division between Clusters 1–3, which dominate western Europe and exhibit a nested concentric pattern, and Clusters 4 and 5 which are almost entirely internally contiguous and sit longitudinally adjacent to one another. These two groups of clusters are neatly divided by the border of the former Eastern Bloc, and distinguished by two key factors: degree of internal variance, and mortality timeline (see Sect. 3.3).

### The clusters display distinct mortality trajectories

Figure 2 shows the overall estimated excess mortality within the identified clusters, and how the trajectories developed during 2020 and 2021 (see also Table  1 for the statistical summary). Four major mortality waves are evident, although not every wave occurs in every cluster. Total excess mortality over the two-year period is not a strong prediction of cluster affiliation ($\chi ^2 = 769, \, \text {df} = 768, \, p = 0.483$; see Fig. 6 in see Appendix Cumulative excess mortality by cluster). Instead, it’s the distribution of deaths along the time dimension that makes the clustered trajectories distinct.

Cluster 1’s trajectory peaked at 352% excess mortality in the last week of March 2020, and Cluster 2’s at 113% one week later. Cluster 3 did not experience any such spikes, although elevated rates of excess mortality are evident: it reached 22% excess mortality during the first wave in March 2020, and peaked at 27% during the second, after which excess mortality in this cluster never exceeded 20% in any single week again.

After the initial surge, Cluster 1 didn’t exhibit another major mortality wave, only reaching 25% excess mortality during the second wave at the end of 2020. However, unlike Cluster 1, Cluster 2 did go on to experience a major second mortality wave, reaching 67% excess mortality in the first week of November 2020. This second wave coincided with the first major (96%) excess mortality peak in Cluster 4, although there is almost no geographical contiguity between them. Cluster 4 experienced two further waves in 2021, reaching 65% in a spring surge at the end of March, and 72% in the last week of November. Like Cluster 4, Cluster 5’s first mortality wave came at the end of 2020, although the peak of this wave trails Cluster 4’s by a few weeks, reaching 88% in the first week of December. Cluster 5 experienced the same springtime surge in 2021 as Cluster 4, hitting 50% excess mortality in the last week of March, then an earlier and more intense wave in October at which points it peaked at 106%. Cluster 5’s dynamics differ from Cluster 4 in that its first wave was less deadly than its third.

**Table 1:**
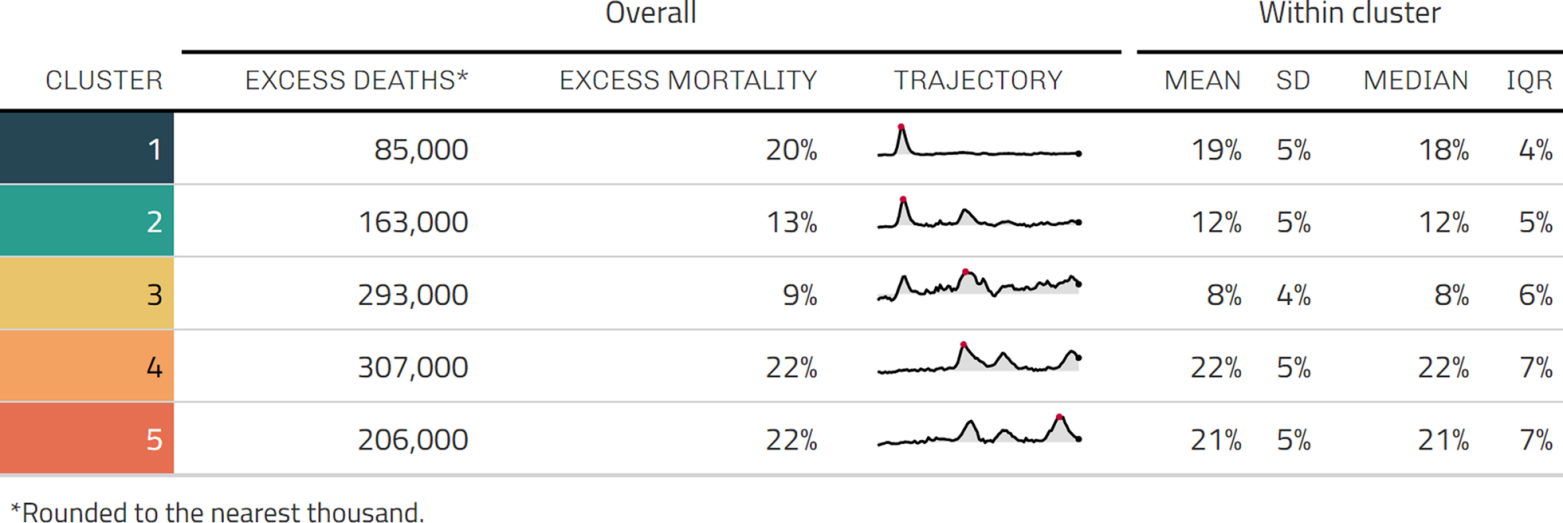
Overall excess mortality, expressed as percentage and rounded estimate, for groups of NUTS3 administrative units of Europe clustered on excess mortality time series trajectories for the pandemic years 2020–2021

### Two subgroups are defined by contrasting characteristics

As noted in 3.1, there are two subgroups evident in the clustering result, which are differentiated by key spatial and temporal characteristics and separated from one another by the Cold-war era border dividing Western Europe from the Eastern Bloc. The two groups are characterised by the contrast in the internal spatial arrangement of their clusters, and by the timeline along which they experienced the pandemic.

**Fig. 3:**
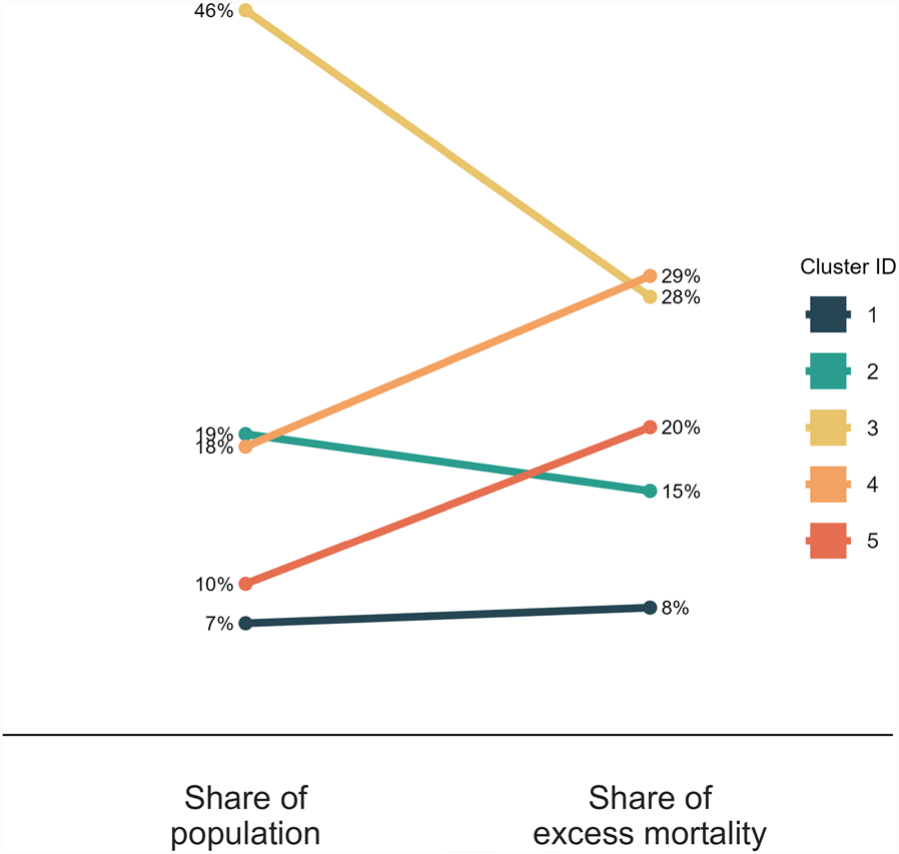
The share of the population vs the share of overall excess mortality in NUTS3 administrative regions of Europe clustered on their excess mortality time series trajectory for the pandemic years 2020–2021. Clusters 4 and 5 represent a disproportionately large share of excess mortality relative to their share of the population

Clusters 1–3 cover the area to the west. They are arranged in a concentric pattern, with heavily impacted Cluster 1 units, which experienced only one abrupt first wave, surrounded by Cluster 2 units, where a (comparatively) less severe first wave was followed by a second wave that winter. The remainder of the western European space is assigned to Cluster 3, which experienced no waves of comparable severity but still contributed massively to the overall mortality toll due to the large population it represents.

The area to the east of the former Iron Curtain exhibits far more spatially homogeneous dynamics. In contrast to the concentric arrangement of cluster groups in the west, clusters 4 and 5 are almost entirely internally contiguous and separated from one another by Hungary’s southern border. In this area, the cluster groups are far more uniform within and across blocs of contiguous states. Not even major cities exhibit the sort of exceptional dynamics that characterise Cluster 1, and no area remains as little affected as Cluster 3. Instead, three consecutive waves reaching 80–100% excess mortality sweep the entire region, in a stark contrast to the concentric hotspot dynamics that characterise the West.

The other key difference between the two subgroups is their contrasting timelines. The western subgroup experiences its first wave in the pandemic’s first spring (i.e. March 2020); the eastern subgroup experiences its first wave in the winter of 2020–2021. In both subgroups, three pandemic waves are evident, but they are offset by eight months in the clusters to the East.

### Absolute numbers show the relative impact of contrasting pandemic dynamics

Absolute, as opposed to relative, excess mortality estimates put the wave dynamics and ‘hotspot’ outliers into perspective (see Table 1). Note that these estimates are a relatively crude indicator prioritizing pan-European comparability and do not represent actual counts. They are valuable in that they give a sense of the mortality impact’s contrasting magnitude between cluster groups, but should be taken as indicative.

The 20% overall excess mortality rate recorded in Cluster 1 represents more than 85,000 excess deaths. In contrast, although Cluster 3 registered a lower overall excess mortality (9%) compared to the other clusters, its large total population means that this represents an extremely large number of people: an excess of 293,000 deaths distributed over two years of sustained elevation in all-cause mortality.

Clusters 4 and 5, which both sustained the same excess mortality rate over the entire time period (22%), account together for over half a million excess deaths (see Table. 1).

Considering the time dimension and population size gives additional context to these estimates and further highlights the contrast in pandemic dynamics between clusters.

While the 293,000 excess deaths in Cluster 3 took the entire two year period to accumulate, 74% of the 85,000 excess deaths in Cluster 1 occurred in just 10 weeks, between March 3 and May 11 2020. Figure 3 shows the share of the population vs the share of excess mortality which accrue to each cluster. Clusters 4 and 5 account, cumulatively, for only about 28% of the total population of the areas of Europe which were included in this study, yet sustained a full half of the excess mortality impact. The delay until November 2020 of the first wave in Clusters 4 and 5 makes the impact of the pandemic in these regions even more disproportionate. The temporal offset concentrates their elevated mortality burden within a shortened timeframe: 97% of the excess mortality impact in Clusters 4 and 5 was incurred after October 10 2020.

## Discussion

Clustering weekly excess mortality time series at NUTS3 level identifies distinct pandemic trajectories which make the spread of the pandemic’s overall mortality burden throughout Europe during 2020 and 2021 more interpretable. The analysis improves upon previous cluster analyses of Europe’s COVID-19 experience by taking excess mortality as the dependent variable, covering a broader geography and a longer timeline, and using higher resolution data [11, 12, 37, 41, 52].

This approach identifies groups of subnational regions with similar trajectories, revealing internal variance within states, and cross-border similarities that indicate shared experiences across Europe. As an alternative to high-level territorial aggregations, the cluster groups present a solution to aggregation bias and distortion that maintains and enhances interpretability of noisy, granular data.

These results confirm and connect the asymmetric pandemic impacts previously described in the literature, particularly the temporal offset and inversion in spatial dynamics between western and eastern Europe. They corroborate and clarify Meintrup et al.’s [41] observed ‘flip effect’ of elevated mortality levels from west to east between the first and second waves, and further develop these findings at higher resolution. The clusters differentiate spatially asynchronous mortality waves (as described by Harvey et al. [28]) at the subnational level, revealing the contrast between western Europe’s concentric mortality patterns and eastern Europe’s relative spatial consistency.

The contrasting dynamics between the western and eastern subgroups offer a broad, consolidating context for more localised and time-limited studies that reconciles seemly conflicting conclusions. For instance, Rangachev et al. [46] observe in their subnational analysis of excess mortality impact in Bulgaria that major cities such as Sofia experienced less severe mortality outcomes than peripheral regions, unlike initial outbreaks in western Europe which were concentrated in densely populated areas [43]. Placing Bulgaria’s experience in the context of the overarching Cluster 5 trajectory, we see that the lack of major urban pandemic hotspots and relative internal consistency in comparison to western European countries is consistent with the pandemic experience of the entire Cluster 4-5 bloc.

COVID-19’s staggered arrival in communities across Europe, and the timing of non-pharmaceutical intervention (NPI) responses, may explain the dynamic juxtaposition between the western and eastern cluster subgroups. Multiple analyses share the conclusion that the degree to which the virus had penetrated communities by the time NPIs were enacted had a significant impact on mortality outcomes [2, 29, 45, 51]. Whether the virus genuinely arrived earlier in Italy, Spain, France, and Belgium than in Poland, Bulgaria, Hungary and Romania, or whether it was only detected earlier, is impossible to prove. The virus was already circulating in Italy and France some weeks before it was detected [14, 16, 22, 36]; there’s no reason why the same may not be true elsewhere. However, in the areas covered by Clusters 4 and 5, most countries had enacted some form of social contact restriction before community circulation (defined by Ylli et al. [55] as the date at which 100 cases were confirmed) had taken hold (see Fig. 8) [26]. This may explain both the 8 month temporal offset of the first waves between west and east and the contrast in spatial mortality dynamics.

The concentric, asynchronous wave dynamics of the western subgroup (Clusters 1-3) are consistent with spatially contagious diffusion patterns, with impacts radiating outwards from epicentres. These epicentres are assigned in our results to Cluster 1. All of these Cluster 1 units are associated with at least one identified superspreading event in the days immediately prior to the WHO’s declaration of a pandemic on March 11 [3, 7, 18, 22–25, 30, 39]. Superspreading plays a prominent role in COVID-19 transmission; a small number of people cause a large proportion of new cases, and public events during which people who would not routinely meet share physical space are extremely effective spreaders, creating long tails of secondary infections throughout the community [4, 15, 48]. By the time the WHO declared a pandemic on March 11 2020, *per capita* confirmed cases in areas assigned to Cluster 1 were already almost 10 times higher than in the rest of Europe [42].

In contrast, the spatially uniform surge in deaths across the clusters which comprise the eastern subgroup points to a confluence between a pre-dispersed virus and changing seasonal conditions. Not limited to localised outbreaks, the pandemic’s delayed first wave in Clusters 4 and 5 allowed for 8 months of stealthy summertime spread during the warmer months when reproduction rates fell and NPIs were tentatively relaxed.

Having identified and described the spatially asynchronous waves and distinct geographical clusters that characterise COVID-19’s mortality impact in Europe, we can use this insight as a basis for more exploratory and explanatory research. For instance, we might ask:

What can we learn from comparing subsequent waves within cluster groups? The results of this analysis identify appropriate geographies for comparison over time, at scale, to answer questions such as:

- Why did Cluster 2 areas experience such a significant second wave, when the Cluster 1 hotspots did not? Can this be explained by the drop in susceptible population, or in differences in population behaviour?
- Do subsequent waves in comparable places affect different demographics over time (as suggested by Marí-Dell’Olmo et al. [38]), and if so how does this impact the efficacy of NPIs?
- What role does the emergence of new variants play in subsequent waves? How do variants’ differing severity and transmissibility affect mortality impact in successive waves?

What can we learn from the contrasting dynamics between the western and eastern subgroups? The division in European mortality patterns poses questions like:

- Why did the resumption of NPI measures fail to protect the population of Clusters 4 and 5 in the autumn of 2020, after the period of summer relaxation? Can the breakthrough surge in cases which led to the eastern subgroup’s first wave be attributed to its dispersal throughout the population, the characteristics of a later variant, changes in population adherence to NPI measures, or some other factor?
- What can we learn about risk and mitigation by comparing the distribution of deaths in the two subgroups?
- What role does the structural legacy of geopolitical history play in shaping Europe’s COVID-19 outcomes? Is the division between the subgroups along Cold-war-era lines meaningful? What role do the contrasting economic, social, and demographic features of the two groups play?

Our analysis uses raw death counts, and takes the mean of the previous five years as its baseline mortality estimate for each region. There are some major limitations associated with this approach. Given the differing risk profile for COVID-19 between age groups, we acknowledge that without age stratified data we are unable to account for the increased risk to older age groups. This limits the interpretation of our results to overall mortality burden; it cannot be taken to indicate relative risk between regions.

**Fig. 7:**
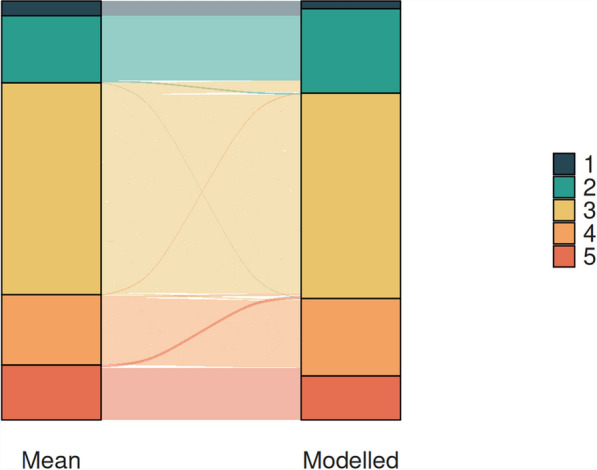
Comparing clustering result between excess mortality estimates based on the mean of the previous five years, versus estimates generated by a Poisson regression model

**Fig. 8:**
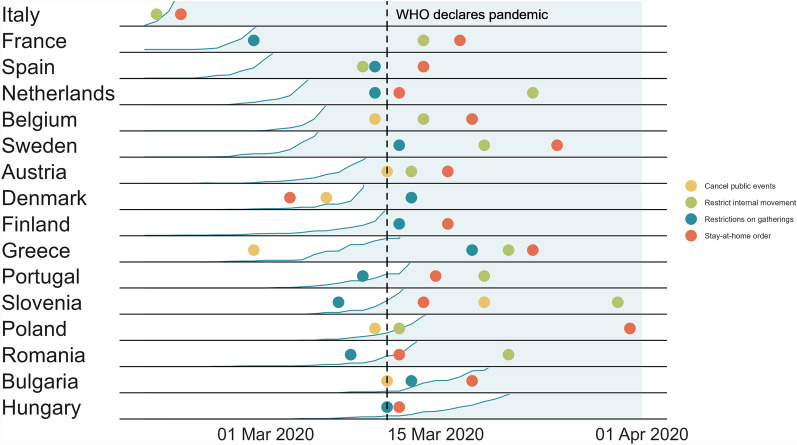
Timeline community transmission onset (represented by 100 confirmed cases, y-axis scale: 0–100), and introduction of restrictions on public events, gatherings, and internal mobility [17, 26]

We chose to limit the analysis to raw death counts on the basis that, if inconsistencies exist between countries in how deaths, population figures, or demographic characteristics are recorded or interpreted, relying solely on historical mortality data from the same source provides greater internal consistency and comparability than attempting to standardize rates using potentially heterogeneous or incomplete external data. However, this approach has its own limitations. It does not account for changes in population, nor for mortality trends in the preceding years. We performed a secondary analysis to manage this limitation, and found that an alternative method to estimate excess mortality using mortality trends did not meaningfully alter our results (see Fig. 7 in Appendix Alternative baseline estimates).

No formal uncertainty estimates were generated for either the excess mortality or the clustering results. This is due to our use of the AP clustering method, which is deterministic and does not accommodate uncertainty. In the absence of uncertainty quantification, we include a robustness assessment using two alternative methods (*k*-NN and hierarchical clustering), which showed broadly consistent results (see Appendix Method comparison and robustness assessment).

A key limitation of the study is Germany’s absence from the all-cause mortality dataset. This is particularly unfortunate given Germany’s central position in the geography and history of Europe, since the inclusion of this data could enable a much more nuanced investigation of the different dynamics between Clusters 1–3 and 4–5 along the border of the former Federal and Democratic republics. Eurostat’s all-cause mortality dataset is built from data routinely collected by state statistical agencies. Germany does not maintain a central repository for civil records, and only annual data are routinely collected at NUTS3 level by the Statistisches Bundesamt via the devolved reporting system [49]. We attempted to include Germany in this analysis by using NUTS2 data (i.e. Federal States), but found that the disproportionately large units introduced aggregation bias. Reluctantly, we excluded Germany from the analysis.

Another limitation is the lack of reliable data relating to causes of death. Taking raw excess mortality as the dependent variable is a double-edged sword. On one hand, it’s a comprehensive indicator which overcomes the detection and definition challenges which plague analyses of COVID-19 case and death rates, and incorporates the pandemic’s broad impacts into a single variable. On the other hand, it doesn’t allow us to separate the causes of death, so this analysis cannot tell us how much excess mortality is directly attributable to deaths from COVID-19, and how much is attributable to incidental deaths from accidents or barriers to medical treatment for routine health problems which might arise when health systems are overloaded with COVID-19 cases.

For the purpose of interrogating different administrations’ approaches to protecting public health during the COVID-19 pandemic, we would need to be able to differentiate causes of death. However, there is no international standard for recording COVID-19 deaths. The variability even within Europe regarding how deaths with or from COVID-19 were classified and counted is too great to allow meaningful intercomparison. For this reason, excess mortality drawn from raw death counts is the better indicator in this instance.

## Conclusion

We set out to explore and describe the spatiotemporal dynamics of the COVID-19 pandemic in Europe at a higher resolution and with better quality data than existing research. We identified five distinct excess mortality trajectories that map to spatial patterns, revealing significant differences in pandemic dynamics between western and eastern Europe. In western Europe, COVID-19’s mortality impact spread outward from a few key epicenters in a ripple effect. Eastern Europe, where the first major wave of excess mortality occurred eight months later, experienced a more spatially homogenous and overall more severe mortality impact. Further research should explore the underlying factors contributing to these divergent patterns and assess how different risk factors or policy responses may have influenced these outcomes.

## Figures and Tables

**Fig. 4:**
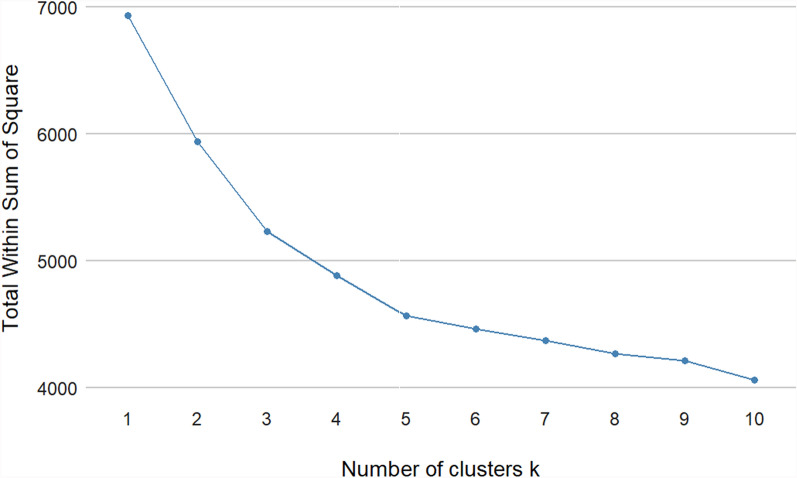
The elbow method for establishing the optimal *k*-value for *k*-NN clustering of 772 NUTS3 units

**Fig. 5:**
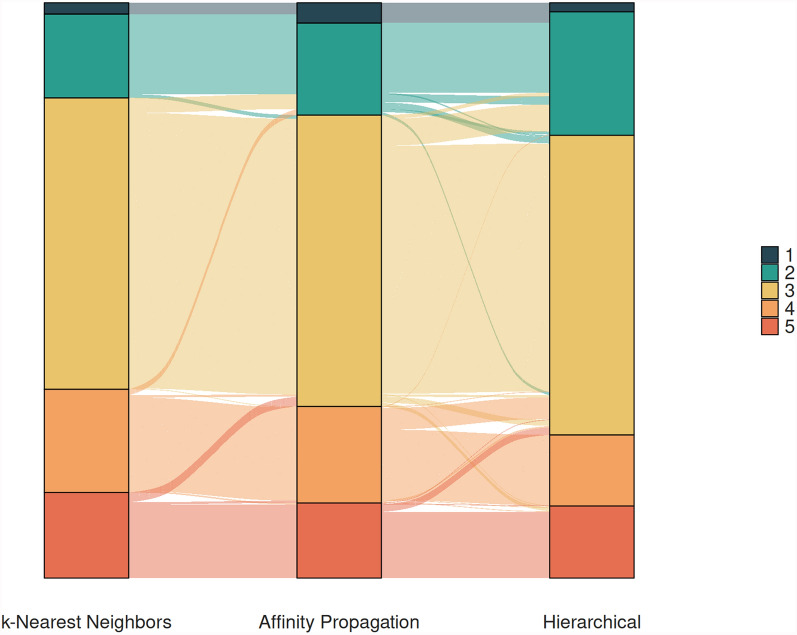
Comparison of cluster assignments of the same 772 NUTS3 units between two clustering algorithms, *k*-Nearest neighbors and affinity propagation

**Fig. 6:**
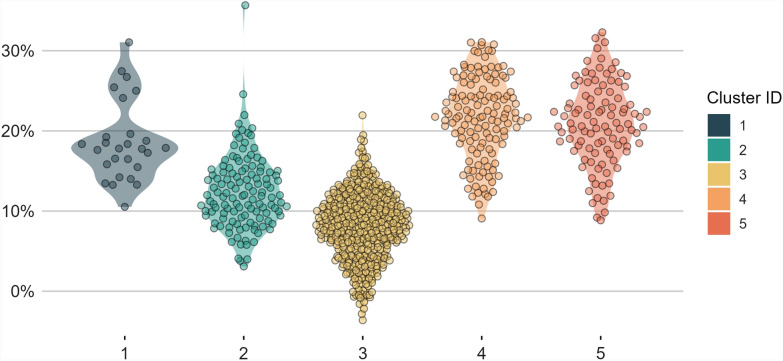
NUTS3 units of Europe, clustered by excess mortality trajectory, by total excess mortality 2020–2021 and cluster affiliation

## Data Availability

This study was conducted using the deaths by week – special data collection (demomwk) dataset which is collated and published by Eurostat. It can be accessed at the following URL: https://ec.europa.eu/eurostat/cache/metadata/en/demomwk_esms.htm

## Appendix

### Method comparison and robustness assessment

We compared the outputs of three clustering methods, and concluded that while the clustering dynamics were broadly consistent across all methods, AP offered the most useful and intuitive output.

We used the Within-Cluster-Sum of Squared Errors (WSS) to establish the optimal *k* value for clustering with *k*-NN. The WSS measures the variance within each cluster, indicating the compactness of clusters. The within-cluster sum of squares (WSS) measures the variance within each cluster, indicating the compactness of clusters. The ideal cluster count is at the “elbow” point (see Fig. 4) where adding more clusters results in diminishing returns on reducing the WSS.

Figure 5 shows the outputs of our three clustering approaches: *k*-NN, AP, and hierarchical clustering with Ward’s method. Overall the results retain the same structure with only minor changes between the clusters. 67 units are reassigned between *k*-NN and AP. 124 are reassigned between AP and the hierarchical method.

### Cumulative excess mortality by cluster

We tested the relationship between overall excess mortality and cluster affiliation, and found that it was not statistically significant ($\chi ^2 = 769$, $\text {df} = 768$, *p* value 0.483). Figure 6 shows the distribution of NUTS3 units by total excess mortality across cluster groups.

### Alternative baseline estimates

Because we do not account for mortality trends in our approach, regions with notable population growth or decline may show biased excess mortality estimates. About 8% of the NUTS3 units in our analysis exhibit a statistically significant temporal trend in raw death counts. To manage this limitation, we performed a secondary analysis, using a Poisson regression model based on the approach used in Ackley et al. [1] to model the expected death count based on past trends. This did not meaningfully alter the results: 67 NUTS3 units were reassigned, but the overall dynamics of the results remained consistent.

### The pandemic onset timeline

This figure shows the timeline to onset of community transmission, represented by 100 confirmed cases, and the introduction of selected key containment measures (Fig. 8).
